# E3 Ubiquitin Ligases Neurobiological Mechanisms: Development to Degeneration

**DOI:** 10.3389/fnmol.2017.00151

**Published:** 2017-05-19

**Authors:** Arun Upadhyay, Vibhuti Joshi, Ayeman Amanullah, Ribhav Mishra, Naina Arora, Amit Prasad, Amit Mishra

**Affiliations:** ^1^Cellular and Molecular Neurobiology Unit, Indian Institute of Technology JodhpurJodhpur, India; ^2^School of Basic Sciences, Indian Institute of Technology MandiMandi, India

**Keywords:** E3 ubiquitin ligases, neurodevelopmental disorders, neurodegenerative diseases, neurons, aging

## Abstract

Cells regularly synthesize new proteins to replace old or damaged proteins. Deposition of various aberrant proteins in specific brain regions leads to neurodegeneration and aging. The cellular protein quality control system develop various defense mechanisms against the accumulation of misfolded and aggregated proteins. The mechanisms underlying the selective recognition of specific crucial protein or misfolded proteins are majorly governed by quality control E3 ubiquitin ligases mediated through ubiquitin-proteasome system. Few known E3 ubiquitin ligases have shown prominent neurodevelopmental functions, but their interactions with different developmental proteins play critical roles in neurodevelopmental disorders. Several questions are yet to be understood properly. How E3 ubiquitin ligases determine the specificity and regulate degradation of a particular substrate involved in neuronal proliferation and differentiation is certainly the one, which needs detailed investigations. Another important question is how neurodevelopmental E3 ubiquitin ligases specifically differentiate between their versatile range of substrates and timing of their functional modulations during different phases of development. The premise of this article is to understand how few E3 ubiquitin ligases sense major molecular events, which are crucial for human brain development from its early embryonic stages to throughout adolescence period. A better understanding of these few E3 ubiquitin ligases and their interactions with other potential proteins will provide invaluable insight into disease mechanisms to approach toward therapeutic interventions.

## Introduction

Development of brain is a long, self-oriented, tightly regulated, complex molecular process governed by various crucial genes, linked with cell proliferation and differentiation (Gilbert et al., 2005). Previous findings have revealed the role of neural stem cells and several other genes in different neurobiological stages of brain development, such as differentiation of the neural progenitor cells, neural tube formation, neural patterning, neurogenesis to neuronal migration and neuronal myelination (Stiles and Jernigan, 2010; Gage and Temple, 2013). But, how these genes and their relevant end product proteins are stringently regulated under the entire span of neurodevelopment is not well understood. Previous studies suggest that E3 ubiquitin ligases play a pivotal role in different neurodevelopmental stages (Huang, 2010; Stegmuller and Bonni, 2010). Several other genes share and regulate the overall burden of neurogenesis and brain development (Preuss, 2012). Mutations or genetic disturbances in these genes generally affect the cognition and behavior, which may also represent the functional loss of crucial cellular processes, such as, synaptic functions, protein translation, cellular proliferation and differentiation (Ernst, 2016). Continuous cellular and molecular deficits of these neurobiological mechanisms are noticeable, which may result in several neurodevelopmental disorders (Sahin and Sur, 2015).

Eukaryotic cell evolution is one of the greatest landmarks in the history of life, marking the formation of hyper-structures and ultra-specialized cellular systems, which are probably developed from the earlier simpler forms of organisms, i.e., prokaryotes (Norris and Root-Bernstein, 2009; Archibald, 2015). These cells perform a variety of cellular tasks with utmost accuracy; and while doing so, cells need a large number of proteins with varying shapes, sizes, subcellular locations, and functions (Zimmerberg and Kozlov, 2006; Ananthakrishnan and Ehrlicher, 2007). Although cells contain around 20,000 different kinds of protein-coding genes, they still produce and retain only a set of proteins at a time, from all the available sequences, in accordance with the requirements of the cells (Kim et al., 2014; Wilhelm et al., 2014). Furthermore, cells need a tight regulation of synthesis and degradation of proteins, without which successful execution of cellular functions is not possible (Gallastegui and Groll, 2010). Cells comprise a subset of approximately 1400 specialized proteins in their repertoire, which is essentially required to achieve and maintain a functional state of its proteome (Balchin et al., 2016). A large number of chaperones and their cofactors assist newly synthesized linear polypeptide chains in attaining their functional three-dimensional shapes by intercepting unfruitful inter-domain interactions and protect them against various kinds of cytotoxic stresses, in order to prevent unwanted misfolding events inside the crowded cytoplasmic *milieu* (Ellis, 1987; Hartl and Hayer-Hartl, 2009; Hartl et al., 2011). Defying such kind of tight control, a subset of proteins still remains structurally disordered in the cell, and are further taken care by molecular chaperones (Walter and Buchner, 2002; Dunker et al., 2008).

Age-related changes and continuous stresses cause a significant decline in efficiency of molecular chaperones, which may result in accumulation of proteinaceous aggregates inside the cells (Tyedmers et al., 2010). Such conditions may result in progression of various types of cancers and neurodegenerative diseases (Morimoto, 2008; Kim et al., 2013). To avoid such unwanted deleterious changes, chaperones may also opt to degrade accumulated toxic proteinaceous burden of the cell, in concerted mechanisms, carried out by cellular proteolytic systems, viz., autophagy and UPS (Arndt et al., 2007; Hartl et al., 2011). Protein degradation machinery of the cell facilitates the degradation of cellular proteins, which have greatly varying half-lives, ranging from few minutes to several hours (Bachmair et al., 1986; Belle et al., 2006; Schwartz and Ciechanover, 2009). Both of these pathways recognize small ubiquitin molecules attached to cellular proteins (the process is called ubiquitylation), as the tags of death, and initiate the degradation pathways. Ubiquitin is a very small protein of 76 amino acids in length, and approximately 8.5 kDa of molecular weight (Ciehanover et al., 1978; Glickman and Ciechanover, 2002; Ciechanover, 2005).

**FIGURE 1:**
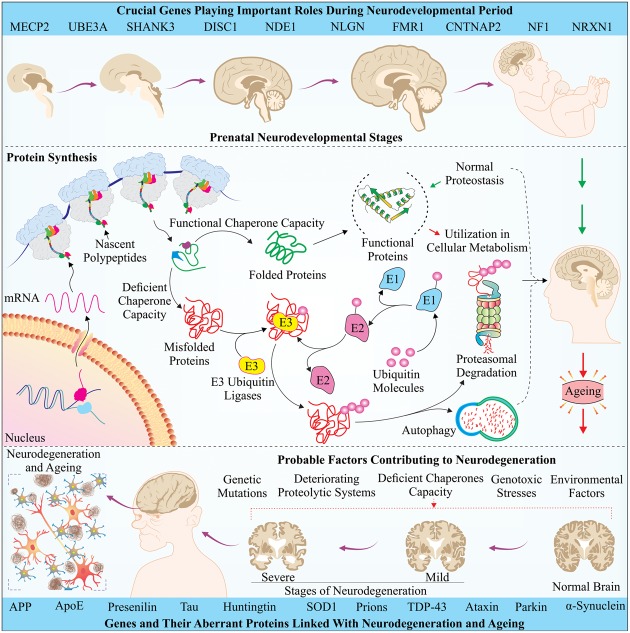
**An illustration of the orchestration of factors involved in neurodevelopment and neurodegeneration:** The upper part of the figure represents how stepwise modifications occur during the prenatal period of brain development. As described in respective sections of the text, few crucially important genes, which play pivotal roles at various stages of brain development, have been mentioned at the top. To establish a better understanding, schematic of cellular translation machinery along with other PQC systems has also been drawn. A state of proteostasis and active control of PQC system regulate the processes of growth, adolescence, and development. However, aging related metabolic changes, including various kinds of stresses and successive deterioration of quality control systems may lead to accumulation of several proteins, causing the formation of inclusion bodies, which results in late-stage neurodegeneration. Few important established protein candidates, involved in various such diseases have been mentioned at the bottom panel; for details, please refer text.

Ubiquitylation is a type of post-translational modification of a protein, in which a concerted action of multiple players, lying into a cascade of reactions, attaches a small ubiquitin moiety to a substrate (Hershko et al., 1983, 2000). It may lead either to a functional modulation, or it may also be treated as death signals, depending upon the pattern, the ubiquitin molecules are attached (Woelk et al., 2007; Komander and Rape, 2012). As illustrated in **Figure 1**, the original discoverers have identified a series of enzymatic reactions, in which, the formation of a thioester bond between C-terminal glycine residue of ubiquitin and a cysteine residue present on E1 ubiquitin activating enzyme activates the ubiquitin in an ATP-dependent manner (Haas and Rose, 1982). Thereafter, the activated ubiquitin is transferred to another cysteine residue, present on a different class of enzymes, called E2, through transesterification (Hershko and Ciechanover, 1992; Ciechanover, 1994). Afterward, transfer of activated ubiquitin to the target protein is mediated by a large group of another set of enzymes, known as E3 ubiquitin ligases (Hershko et al., 1983). Increasing reports suggest that the number of E3 ubiquitin ligases is reaching around thousand, which enables them to provide substrate specificity inside the cells to take control of most of the major and minor cellular pathways (Nakayama and Nakayama, 2006).

These enzymes attach the ubiquitin molecule to the 𝜀-amino group of an internal lysine residue of the substrate protein, or in other cases, to one of the seven lysine residues present on an already attached ubiquitin molecule (Ciechanover, 1994; Hershko et al., 2000). The way, the number, and the pattern, into which the ubiquitin moieties get attached to a given protein, provide the code, on which other cellular systems act and decide their diverse fates, as they could be sorted, trafficked, functionally or structurally modulated or degraded (Rotin and Kumar, 2009; Rajalingam and Dikic, 2016). After finding a suitable degradatory signal, the proteolytic machinery of the cell, the 26S proteasome degrades the substrate into smaller oligopeptides (Finley, 2009). However, to prevent such degradation signals from being constitutive, cells need to remove these ubiquitin chains immediately from the substrate proteins, once they have been utilized. For this, cells employ another set of approximately a 100 different deubiquitinating (DUBs) enzymes, which by shredding off ubiquitin moieties from proteins, contribute largely to their proper recycling (Komander et al., 2009; Reyes-Turcu et al., 2009). Therefore, similar to kinases and phosphatases, E3 ubiquitin ligases and DUBs also provide a mechanism of reversible modifications of cellular proteins to alter their functions (D’Andrea and Pellman, 1998; Dikic and Robertson, 2012).

Roles of E3 ubiquitin ligases have been recognized in the developing, maintaining, as well as in degrading the components of the cells. Developing roles include those, which are played by E3 ubiquitin ligases in developmental processes, such as, cell division, stem cell differentiation and organogenesis, etc. (Nakayama and Nakayama, 2006; Yokomizo and Dzierzak, 2008; Stegmuller and Bonni, 2010). Maintenance responsibilities include, cell signaling, metabolism, transcriptional control, protein sorting, trafficking, cell to cell communication and modulation of inflammatory responses, etc. (Liu, 2004; Weake and Workman, 2008; Acconcia et al., 2009; Hampton and Garza, 2009; Huang, 2010; Polo, 2012). However, E3 ubiquitin ligases are also profoundly associated with the establishment of cellular proteostasis by regulating the turnover of cellular proteins, using degradatory pathways of UPS and autophagy (Schwartz and Ciechanover, 2009; Kuang et al., 2013). Induction of apoptotic pathways could also be attributed to degradatory functions of the E3 ubiquitin ligases (Vucic et al., 2011). Continuous degradation of intracellular proteins via proteasome or autophagy also facilitates recycling of amino acid pool of the cells for synthesis of new proteins (Lilienbaum, 2013).

It is well accepted that brain development is one of the most complex biological processes, which starts in early gestation period and lasts up to adolescence, and is orchestrated and affected by both genetic and environmental factors (Lenroot and Giedd, 2008; Stiles and Jernigan, 2010). Several billion neurons integrate together to form a network called neural circuitry, which receives and transmits electrochemical signals in order to perform cognitive and behavioral functions with regulatory control (Pakkenberg and Gundersen, 1997; Tau and Peterson, 2010). Therefore, to completely understand the process of neurodevelopment and to address the problems of developmental abnormalities, representation of a comprehensive overview of the majorly associated environmental and genetic factors is needed. We, here, are providing a brief outline of how several external stresses, pollutants, toxic chemicals and consumption of certain compounds may obstruct the successful development.

Regulatory control over a large set of processes makes E3 ubiquitin ligases a putative therapeutic target, with huge potential in the upcoming years of research, in the field of developmental disorders, neurodegeneration, ageing, and cancer (Sun, 2006; Eldridge and O’Brien, 2010; Goru et al., 2016). In this review, a comprehensive description of few important E3 ubiquitin ligases, which are crucial for various kinds of developmental processes, has been provided. We have also given a concise overview of few emerging players, which have been evolved in past few years as crucial regulators of various neurodevelopmental and neurodegenerative disorders, for example, ITCH, E6-AP, MGRN1, and HACE1, which certainly need attention of scientific community for their remedial exploration (Rotin and Kumar, 2009; Upadhyay et al., 2015a).

A large number of E3 ubiquitin ligases form an intricate network, over which regulatory mechanisms of several cellular pathways are built. This is why the UPS is considered to be a highly specific degradation mechanism for intracellular proteins, unlike autophagic and lysosomal degradation pathways. As stated earlier, there is increasing evidence clearly indicating the involvement of E3 ubiquitin ligases in tight regulation of most, if not all, of the cellular processes (Hilt and Wolf, 2000; Joshi et al., 2016). These E3 ubiquitin ligases sense various kinds of changes and stresses quickly inside the cytosol, and mount a simultaneous response by affecting the turnover or functionality of cellular proteins, which play regulatory roles in several cellular processes, e.g., different receptors, tumor suppressors, kinases, and transcriptional regulators, etc. (Mayer et al., 2005; Woelk et al., 2007; Polo, 2012). In the subsequent sections, we will briefly provide a summary of research done so far, in the field of both, proteolytic and non-proteolytic ways of regulation of cellular proteins, mediated by a plethora of E3 ubiquitin ligases.

## Neurodevelopmental Processes and Neurodegenerative Diseases are Affected by Environmental and Genetic Factors

Neurodevelopment associated problems have always remained a great challenge to understand for physicians and scientists. Considering research done in last few decades, it would be obvious to say that environment has a deep impact on organism growth, development, evolution, and extinction (Jirtle and Skinner, 2007). Developmental scientists have reported since decades, about how environmental constituents are crucial factors shaping the developmental processes and how environmental stresses and toxicities retard the growth and put challenges before these processes (Knobloch and Pasamanick, 1960). Over the years, it has been observed that prenatal development is a well-programmed process (Huxley et al., 2000) and exposure to various kinds of radiations (Koturbash et al., 2006), xenobiotic chemical compounds (Ho et al., 2006), maternal behavior (Weaver et al., 2004), post-weaning nutrition (Waterland et al., 2006), environmental pollutants (Perera and Herbstman, 2011), and heavy metals (Kampa and Castanas, 2008) have multifactorial deleterious effects on the embryonic health.

Alcohol consumption by the mother during pregnancy may also impair cognitive and behavioral growth (Ornoy and Ergaz, 2010); whereas microcephaly and congenital anomalies are other reported symptoms (Ouellette et al., 1977). Smoking and alcohol intake may also cause ADHD (Mick et al., 2002). On the other hand, increased risks of asthma (Stick et al., 1996) and obesity (Power and Jefferis, 2002) have also been observed in offspring of mothers, who regularly smoke during pregnancy. Regular consumption of marijuana products and drugs like cocaine during gestations period may also have detrimental effects in newly born infants, including lower weight and height (Zuckerman et al., 1989), ADHD and declining cognitive functioning (Huizink and Mulder, 2006). *In utero* exposure to environmental pollutants, e.g., polycyclic aromatic hydrocarbons (Brown et al., 2007), bisphenol A (Ho et al., 2006), arsenic (Smith et al., 2006), and phthalates (Kang and Lee, 2005) could also exert epigenetic hazards, genotoxicity, high risks of lung cancer, and endocrine disruption, etc. (Perera and Herbstman, 2011). There are several other factors and chemical compounds, e.g., oxidative stress, valproic acid, caffeine, lithium chloride, and retinoic acid, which could have many toxic and teratogenic effects on embryonic development (Dennery, 2007; Selderslaghs et al., 2009). But, adding to all these, genetic alterations and mutations constitute the major factors, lying at the bottom of many developmental disorders.

Notably, an increasing line of evidence has also established the confounding roles of a number of genes on developmental processes. These genes could have multiple direct or indirect influences on various pathways and reactions, which are essential for organism development (Wells et al., 2005; Raff, 2012; Zoghbi and Bear, 2012). A well-regulated expression levels of few genes, e.g., p53, BRCA1, Hox, etc., throughout prenatal period are considered to be a crucial factor for development (Choi and Donehower, 1999; Wells et al., 2005; Schuettengruber et al., 2007), whereas mutations reported in other genes have also shown occurrence of some developmental disorders(Mitchell, 2011). Autism is a heterogeneous condition of the spectrum of communication and developmental disabilities in children; it includes several sets of disorders with varying features and commonly known as ASD (Lord et al., 2000; Frith and Happé, 2005).

More than a 100 different genes have been reported so far to be disrupted, deleted or mutated in ASD, developing several kinds of symptoms and disorders like ID and epilepsy (Geschwind and Levitt, 2007; Betancur, 2011; Miles, 2011). Triplet codon CGG expansion and hypermethylation lead to the suppression of FMR1 gene that is a common cause associated with the fragile X syndrome, a well-known intellectual and emotional disorder (Jin and Warren, 2000). Genetic mutations of X-linked genes neuroligin-3 and -4 (NLGN3 and NLGN4) have also shown similar kinds of symptoms leading to autistic disorders (Jamain et al., 2003). CNTNAP2 (Alarcon et al., 2008), neurexin-1 (NRXN1) (Ching et al., 2010) are two other genes, which have been investigated in recent years for their wider implications in various kinds of developmental processes and association with these diseases. Another autistic condition, neurofibromatosis type 1 (NF1), is an autosomal dominant neurodevelopmental disorder, mainly caused by mutations in gene NF1 (Wallace et al., 1990).

Rett syndrome is an X-linked dominantly inherited, autistic neurodevelopmental disorder leading to mental retardation in early childhood (Rett, 1966; Chahrour and Zoghbi, 2007). Reports suggest that the disease occurs due to mutations in MECP2 (Amir et al., 1999). PMS, a global developmental delay related disorder, is found to be associated with disruption of SHANK3 (Bonaglia et al., 2001). Similarly, DISC1 and 2 are other genes, which have been reported to cause a well-known psychiatric mental disorder SCZ, with reported symptoms of anxiety and depression (Millar et al., 2000). NDE1 plays essential roles in mitosis and microtubule arrangements, so have crucial implication in neurodevelopment (Bradshaw Nicholas et al., 2013). NDE1-null mice have been reported recently to develop smaller brain, with cerebral cortex affected mostly (Alkuraya et al., 2011; Ramalingam et al., 2011); whereas single nucleotide mutations in this gene may also result in SCZ like symptoms (Kimura et al., 2015).

The past decades have seen tremendous advancements in health care leading to better health management and hence people living longer. The increase in longevity has led to increased prevalence of neurodegeneration in the elderly people. The risk factors for neurodegenerative diseases could be both, endogenous or exogenous; but have not been well described (Cannon and Greenamyre, 2011; Kanthasamy et al., 2012). Most of the neurodegenerative diseases occur at a late-age, either because of several genetic mutations, or aging-associated decline in cellular repair mechanisms (Finch and Tanzi, 1997; Niccoli and Partridge, 2012; Yerbury et al., 2016). These detrimental changes include alterations in various components of the cellular PQC system, e.g., declined chaperone capacity, weakening proteolytic functioning of autophagy and UPS, and compromised mitochondrial health and ER proteome balance (Germain, 2008; Koga et al., 2011; Kaushik and Cuervo, 2015). Similar to earlier describe developmental abnormalities, environmental factors and various genotoxic stresses also contribute in neurodegeneration and aging processes (Stokes et al., 1999; Cannon and Greenamyre, 2011; Yegambaram et al., 2015). A major hallmark of neurodegenerative diseases is the abnormal intra- or extracellular accumulation of disease-causing aggregate-prone proteinaceous species, which further hinder with the normal functioning of neuronal cells leading to their death, causing cognitive impairment, dementia, and behavioral abnormalities (Kanazawa, 2001; Ross and Poirier, 2004).

As we have described, several genetic mutations lead to developmental deformities or may also cause embryonic mortality in some cases, it is now easily understandable how important genomic integrity is, for the proper functioning of the organism. Several aging-related changes and late-onset neurodegenerative disorders may also add up to the pathologies generated, either due to some genetic mutations, or structural and functional modifications in disease-associated proteins (Bertram and Tanzi, 2005; Esiri, 2007; Douglas and Dillin, 2010). Most prevalent of these, Alzheimer’s disease, has been reported to be caused due to mutations in genes, like APP (Goate and Chartier-Harlin, 1991), presenilins (Scheuner et al., 1996), tau (Hutton et al., 1998), etc. Apolipoprotein E (ApoE), in recent years, have also been shown as one of the risk factors for the disease (Saunders et al., 1993).

Parkinson’s disease is the second most death-causing neurodegenerative disorder and is also linked with several genes viz. PINK1 (Valente et al., 2004), parkin (Kitada et al., 1998), α-synuclein (Polymeropoulos et al., 1997), as disease-onset factors. Prion diseases are another class of diseases, where genetic mutations alter the structure and function of the cellular prion protein, which may develop into the neurodegenerative changes (Prusiner and DeArmond, 1994). ALS is a disease, related to loss of motor neurons, which is caused by aggregation of protein products of several inclusion-forming proteins, e.g., SOD1 (Wong et al., 1995) and TDP-43 (Iguchi et al., 2013). Several proteins may also attain a property to aggregate due to the expansion of glutamine coding CAG trinucleotide repeats in their gene sequences (La Spada et al., 1991). Aberrant forms of huntingtin (DiFiglia et al., 1997) and ataxin (Orr et al., 1993) are major examples of polyglutamine expansion proteins, which are directly associated with the amyloid-like aggregate formation in different diseases.

Neurodegeneration is not a spontaneous process; exposure to various neurotoxicants present in the environment over a period of time leads to irreparable damages. These neurotoxicants interfere with the metabolism and disturb the homeostasis. Heavy metals, herbicides, pesticides, biogenic metals have been described as potential risk factors for neurodegeneration (Brown et al., 2005; Ritz et al., 2009; Chin-Chan et al., 2015). Essential trace elements (aluminum, zinc, and copper) have been found to be associated with aggregated proteins in Alzheimer’s disease (Bush et al., 1994; White et al., 1999; Bharathi et al., 2008). Lead toxicity is also a well-described phenomenon and exposure of children to lead at early developmental stages might influence the occurrence of neurodegeneration, at later stages (Basha et al., 2005). Rural settings with improper sanitation and industrial waste disposal, agricultural runoff, etc., all carry neurotoxicants and pose threat to human beings.

Neurotoxicants can damage or cross blood brain barrier, gain access through receptors having similar ligands (McCormack and Di Monte, 2003). The damage of neurons affects neurotransmission due to lipid peroxidation, which leads to increased ROS generation and oxidative stress (Nunomura et al., 2001; Aruoma et al., 2006; Reed, 2011). The increased oxidative stress results in the formation of protein aggregates, which in turn activates the glial cells. These cells contribute in enhancing inflammation at the site along with the release of NO, leading to further upregulation in oxidative stress, by the formation of ONOO-peroxynitrite (Vincent et al., 1998). The emerging understanding of pathophysiology of neurodegenerative disorders is helping us to understand the potential risk of environmental factors on human health and also come up with novel strategies for therapeutics. Several E3 ubiquitin ligases in past have been explored for their significant contribution in neurodevelopmental processes and involvement in establishment of cellular proteostasis. In the upcoming section, we are briefly describing few similar quality control (QC) E3 ubiquitin ligases and elaborating on how they appear to be silent for a long time interval in life span and how they suddenly become proactive and play a crucial role at the time of requirement against misfolded and accumulated proteins, linked with neurodegeneration.

## Good Programming of UBE3A: How it is Important for Neuronal Development and Neurodegeneration?

E3 ubiquitin ligase E6-AP is encoded by UBE3A gene; mutations and genetic imprinting in this gene result in Angelman syndrome (AS), which can be described by symptoms like frequent laughter, tremor, ataxia, abnormal gait, seizures, and neurological impairments (Kishino et al., 1997). Several studies identified critical neuronal dysfunctions in UBE3A maternal-deficient mice (AS mice model), which include defective synaptic plasticity, neurological deficits, aberrations in LTP, defects in rotarod performance, abnormal dendritic spine morphology, deficits in contextual learning, abnormalities in neocortex maturation, reduced brain weight, cognitive dysfunction and abnormalities in fluid consumption behavior and grip strength (Kuhnle et al., 2013; Silva-Santos et al., 2015). The emerging function of E6-AP were discovered later and is associated with its E3 ubiquitin ligase like capabilities, through which it can play a very important neuroprotective role in cellular QC mechanisms and its functional presence is involved in different neurodegenerative diseases through clearance of several misfolded proteins (Upadhyay et al., 2015a).

Under different stress conditions, E6-AP expression levels are dramatically induced and provide cytoprotection against different proteotoxic insults. E6-AP interacts with Hsp70 and preferentially targets aggregatory proteins for their UPS-dependent clearance. Recruitment of E6-AP with aggresomes at peripheral nuclear regions reveals its capability to recognize large misfolded inclusion-like structures in cells (Mishra et al., 2009b). Considering the efficiency of E6-AP in cellular protein QC mechanisms, it is expected that E6-AP might target neurodegenerative disease-associated proteins. In another study, we have demonstrated that E6-AP facilitates the ubiquitin-mediated degradation of polyglutamine proteins aggregates and alleviates toxicities generated by them in cells. The high expression of E6-AP protects cells against the massive buildup of misfolded proteins that can cause cellular toxicity and finally leads to cell death (Mishra et al., 2008). After understanding the potential of E6-AP against misfolded protein clearance, we again checked its capability in another neurodegenerative disease. We noticed that E6-AP recognizes misfolded SOD1 aggregates and targets them for ubiquitylation and also promotes their degradation via UPS machinery (Mishra et al., 2013).

Previously, we have also found that E6-AP mediates the ubiquitylation and proteasome-dependent degradation of p53 tumor-suppressor protein without E6 oncoprotein (Mishra and Jana, 2008). Lack of E6-AP function results in inefficient elimination of p53, hence may also alter cell cycle progression, and affect the normal physiological functions of different brain regions (Jiang et al., 1998). Interestingly, another study from our group indicates a strong potential of E6-AP in the degradation of cyclin-dependent kinase inhibitor p27. Probably, such type of regulation of p27 and a loss-of-function of E6-AP, both contributes to the molecular pathogenesis of AS (Mishra et al., 2009a). Earlier reports have shown that p27 lack-of-function in knockout mice exhibits increased brain size, which could be possible because of high cellular proliferation rate (Fero et al., 1996; Tarui et al., 2005). E6-AP mediated ubiquitylation and endocytosis of SK2 is implicated in synaptic plasticity and formation of memory and learning (Sun et al., 2015).

**FIGURE 2:**
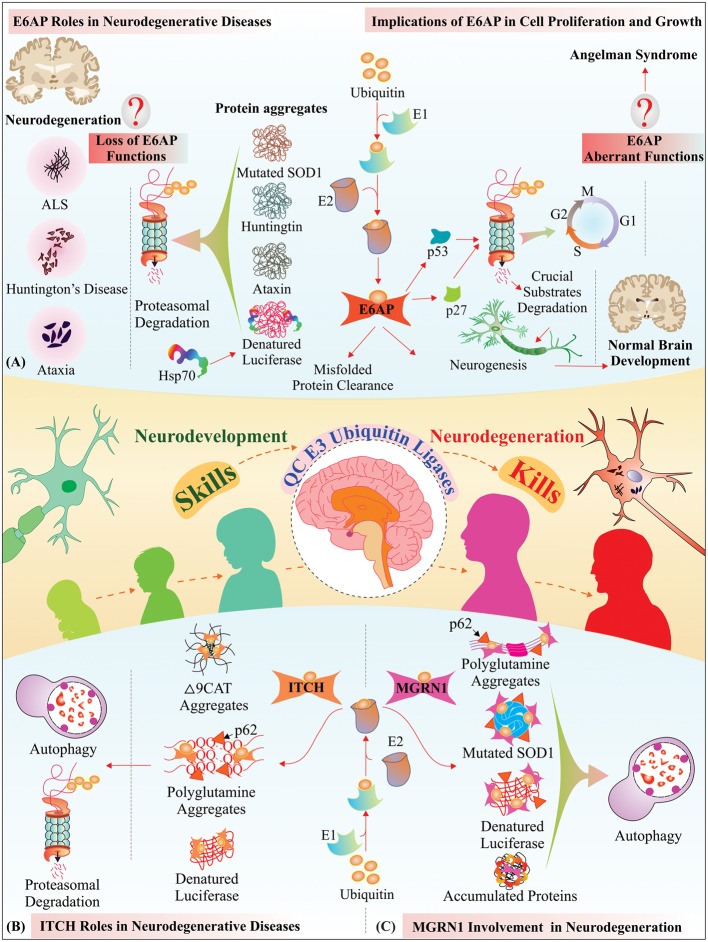
**Schematic representation of emerging roles of neurobiological QC E3 ubiquitin ligases implicated in neurodevelopment, synaptogenesis, and, their lack of functions contribute to the pathogenesis of neurodegeneration:** **(A)** Genetic defects in UBE3A gene results in defective E6-AP protein, which is responsible for Angelman syndrome neurodevelopmental disorder. Functional E6-AP promotes the ubiquitylation of tumor-suppressor proteins such as p53 and p27 and regulates cellular proliferation and growth. E6-AP also plays a significant role in the clearance of mutant misfolded proteins linked with various neurodegenerative diseases. **(B)** ITCH specifically ubiquitylates several cytoplasmic protein inclusions for their removal from the dense crowded cellular milieu. The central segment represents overall functional roles of QC E3 ubiquitin ligases due to their crucial involvement in the neurodevelopment and neurodegeneration. **(C)** Mahogunin functions as a RING finger E3 ubiquitin ligase; and a null mutation in mahogunin is linked with spongiform neurodegeneration. Our studies reveal the potential roles of MGRN1 as a QC E3 ubiquitin ligase involved in the elimination of mutated proteins linked with polyglutamine and ALS neurodegenerative diseases.

E6-associated protein possesses a capacity to regulate cell cycle regulatory proteins, which are vital for cellular proliferation and division. Therefore, E6-AP may contribute significantly in different neurodevelopmental phases, and simultaneously, it can also reduce the accumulation of misfolded proteins in cells and clears the unwanted toxicity generated due to misfolded proteins to avoid neurodegeneration. Interestingly, a finding indicates that AS adults also develop bradykinesia and Parkinsonism symptoms (Harbord, 2001). By truncating or silencing Ube3A-antisense transcript (ATS), improvements in cognition and behavioral functions in AS mice model has been observed (Meng et al., 2013, 2015). Additionally, topoisomerase inhibitor topotecan has also shown to reactivate dormant Ube3a allele having applications in AS therapy (Huang et al., 2011). To summarize and as has been represented in **Figure 2A**, E6-AP in cells provides a pivotal link between NDDs and neurodegenerative diseases. The essential question in near future would be to look up to the molecular functions of E6-AP and to understand how this E3 ubiquitin ligase is capable of switching its neurodevelopmental responsibilities and its QC functions and how it contributes to the alleviation of the pathobiology of neurodegenerative diseases.

## Accurate and Wide Spread Functions of AIP4 Gene Product Orients Neuronal Development and Survival

Atrophin-1-interacting protein 4 gene encodes a HECT-containing ITCH E3 ubiquitin ligase. Loss-of-function of this protein exhibits various immunological problems in mice. ITCH a protein of more than 100 kDa catalyzes the degradation of multiple substrates with the help of UPS (Perry et al., 1998). Regulated protein elimination via autophagy and UPS contributes in different molecular pathways of brain development, e.g., axon guidance, axon and dendritic branching, synaptogenesis and building synaptic connections. Numb protein is important for cell fate determination, differentiation of progenitors of cerebellar granule cells, cell migration and also targets the critical substrates for ITCH and help in their ubiquitylation (Cayouette and Raff, 2002; McGill and McGlade, 2003; Klein et al., 2004). Few fundamental questions always appear in mind, such as what is the physiological relevance of these few specific E3 ubiquitin ligases in neurodevelopmental period?

Currently, we are short of understanding how dysfunctions of E3 ubiquitin ligases or aberrant elimination of unwanted proteins contribute to multiple NDDs and neurodegenerative diseases. To challenge such questions, it was important for us to explore the molecular functions of ITCH under various multifactorial stress conditions, where the massive accumulation of aberrant polypeptides can cause failure of QC mechanisms. For the first time, our study established the QC function of ITCH E3 ubiquitin ligase against cytosolic aberrant aggregates, as has been represented in **Figure 2B**. In our findings, we have noticed that ITCH endogenous levels get elevated after various genotoxic and proteotoxic cellular insults. Sequestosome-1 (SQSTM1) or p62 (early autophagic structures) and 20S proteasome get recruited to the site of the cytoplasmic misfolded aggregate formation with ITCH. Our study also suggests a preferential interactions of ITCH with expanded polyglutamine proteins and further overexpression of this protein reduces aggresomes formation and mitigates the toxic insults generated by expanded polyglutamine proteins (Chhangani et al., 2014b).

Other developmental roles of ITCH are ubiquitylation of JunB for proteasomal degradation to carry out regulation of osteoblasts differentiation from mesenchymal progenitor cells, which may serve as the target of therapies for patients having bone loss (Zhang and Xing, 2013). ITCH also induces Numb-mediated suppression of Hedgehog signaling via ubiquitylation and degradation of transcription factor Gli1 (Di Marcotullio et al., 2006). Deficiency of ITCH has been linked with morphological and developmental abnormalities in addition to multisystem autoimmune disease, as described previously (Lohr et al., 2010). Studies, aiming to understand recognition and interaction of ITCH, may prove to be beneficial in regulating activities of this E3 ubiquitin ligase (Bellomaria et al., 2012; Upadhyay et al., 2015a), which can be further used in studying roles of ITCH in normal metabolism and developing therapeutic interventions against various protein conformational disorders. In the future, it is important to uncover the hidden potential of ITCH E3 ubiquitin ligase, which plays an important role in NDDs as wells as in neurodegenerative diseases. We have to investigate further how the loss-of-function of ITCH contributes to the progression of neurobiological pathomechanism conditions for both NDDs and neurodegeneration. Further, studies should be aimed to solve the problem of aberrant ITCH protein functions and its involvement in neuronal dysfunction.

## MGRN1 Ultra-Compact Neuronal Functions Clean-Up Overload of Unwanted Accumulation of Abnormal Proteins, and Implications in Neuronal Developmental Disorders and Neurodegeneration

A recent review explains the neurobiological functions of another RING finger family member, i.e., MGRN1, an E3 ubiquitin ligase; lack of function of this E3 ubiquitin ligase causes developmental defects such as abnormal left-right axis patterning and congenital heart defects (Cota et al., 2006; Upadhyay et al., 2015b). Previous studies suggest that development of neurons and synaptogenesis are the highly controlled molecular mechanism. MGRN1 expression is also high in both human and mouse brain regions. How does the expression of MGRN1 determine neurodevelopmental events, left-right axis formation and how aberrant functions of MGRN1 cause congenital heart defects are not well-understood? The neurobiological functions of MGRN1 are largely unknown. Growing number of findings indicate the role of MGRN1 in neurodegenerative diseases. MGRN1 null mutants bring much more insights into neuronal functions of MGRN1; loss of MGRN1 causes oxidative stress and mitochondrial abnormalities linked with neurodegeneration (Cheng and Cohen, 2007; Sun et al., 2007).

Mahoganoid mutant mice exhibit the problem of spongiform neurodegeneration. Another study also indicates interaction of MGRN1 with both toxic cytosolic form (cyPrP) and transmembrane isoform linked with prion disease (CtmPrP) (He et al., 2003; Chakrabarti and Hegde, 2009). Interestingly, our recent findings represent cellular QC capacity of MGRN1 under various proteotoxic stress conditions and establish a neurobiological role of MGRN1 against toxic misfolded mutants of SOD1 and expanded polyglutamine proteins, which are involved in causing ALS and polyglutamine diseases, respectively (Chhangani et al., 2014a, 2016). As depicted in **Figure 2C**, recent studies have strongly suggested potential neurobiological roles of MGRN1 in NDDs and neurodegenerative diseases. All the above-summarized studies on MGRN1 show its roles in neurodevelopmental processes and in modulating the QC pathways under different neurodegenerative pathologies (Upadhyay et al., 2015b). These works have paved the way for identifying and characterizing novel E3 ubiquitin ligases, which may act as modulators for neurodevelopmental proteins and probably can also regulate proteostatic pathways, thus would be effectively able to control the occurrence of neurodegenerative disorders. Future research is important to identify the new mechanisms that explore the functions of MGRN1 in brain development, synaptogenesis, synaptic transmission and how the aberrant functioning of this protein contributes to human brain molecular pathophysiology.

## HACE1 E3 Ubiquitin Ligase: Efforts Against Neurodevelopmental Disorders and Neurodegenerative Diseases

Another E3 ubiquitin ligase HACE1, which has an essential implication in NDDs and neurodegenerative diseases, also demonstrates an insight into the relationship between QC mechanism and neurobiological functions. HACE1 encodes a HECT domain-containing E3 ubiquitin ligase involved in the regulation of Rac1 and small GTPases. An exome sequencing analysis identified aberrant functional mutations in HACE1, which leads to autosomal recessive neurodevelopmental disorders with ID, spasticity, and abnormal gait (Hollstein et al., 2015). E3 ubiquitin ligase HACE1, along with UBCH7 E2 enzyme recognizes specific proteins for proteasomal degradation. It also targets Rac1 for polyubiquitylation and regulates its activity. Rac1 dysregulation generates neurodevelopmental abnormalities, whereas an increase in its function exhibits anterior cerebellar deficits, therefore Rac1 is important for defining cerebellar morphology patterning in mice (Torrino et al., 2011; Mulherkar et al., 2014).

Another interesting report indicates that HACE1 promotes the synthesis and stabilization of NRF2 protein, while the lack of function generates oxidative stress. HACE1 expression provides neuroprotective response against toxicity generated by mutant huntingtin protein and interestingly HACE1 level was found to be decreased in Huntington’s disease patients striatum (Rotblat et al., 2014). Pam is an E3 ubiquitin ligase that contains C-terminal RING finger domain and reduction of Pam activity in primary neurons causes downregulation of mTOR signaling and stabilization of tuberin. It is homologous to PHR protein family members and is involved in synaptogenesis (D’Souza et al., 2005; Han et al., 2012). How normal functions of Pam occupy a role in neurodevelopmental phases? Whether lack or aberrant functions of Pam can also aggravate the accumulation of misfolded proteins in cells? These are few unanswered questions for future studies linked with neurobiological functions of Pam E3 ubiquitin ligase. Different reports have shown that E3 ubiquitin ligases, like HACE1 and Pam, are suitable candidates, which take part in neurodevelopment, and may also modulate stress responses via different signaling mechanisms (Han et al., 2008; Rotblat et al., 2014; Hollstein et al., 2015). But, future endeavor based on the existing knowledge of these E3 ubiquitin ligases should focus more on how to effectively utilize this understanding in the development of a suitable therapy for treating disorders, which are affected by these signaling mechanisms.

## E3 Ubiquitin Ligases are Crucial for Brain Development and their Aberrant Functions are Fatal

Most of the studies on E3 ubiquitin ligases are somewhere related to neurodegeneration and associated disorders, which commonly occur at old age (Chhangani et al., 2012; Upadhyay et al., 2015a). In developing brain, different E3 ubiquitin ligases play crucial roles (Stegmuller and Bonni, 2010). They participate in various processes of neuronal development, such as in synapse formation, neurogenesis, neurite enlargement, dendrite growth, axonal development, neural tube formation, and differentiation (Yan et al., 2009; Kawabe and Brose, 2011). Developmental events of neurons depend on various proteins, whose levels are regulated by their respective E3 ubiquitin ligases. Alterations in the levels of these substrate proteins due to loss-of-function or inactivation of E3 ubiquitin ligases have been shown to cause abnormal phenotypic outcomes and may result in neurodevelopmental disorders (Kuhnle et al., 2013; Morice-Picard et al., 2016). Despite being an interesting target, having multiple substrates with involvements in different signaling mechanisms, E3 ubiquitin ligases seem to be difficult candidates to be exploited for therapeutic applications. For example, ITCH suppresses the aggregation of cytoplasmic misfolded proteins (Chhangani et al., 2014b), while it also negatively regulates Hippo pathway that promotes tumorigenicity (Salah et al., 2011).

However, E3 ubiquitin ligase-mediated ubiquitylation and degradation have gained notable importance as an essential part of the early development of eukaryotes, including both animals and plants (Hellmann and Estelle, 2002; Stegmuller and Bonni, 2010). In *Caenorhabditis elegans* cullin RING ligases (CRLs) play an important role for the molecular mechanism of self-renewal and differentiation in embryonic cells during early embryogenesis. SKN-1 transcription factor mediates this differentiation process; and CRLs regulate this crucial molecular switching (Du et al., 2015). In the early *C. elegans* embryo regulation of asymmetry and persistence of the SKN-1 transcription factor also require a defined control, which is provided by a HECT E3 ubiquitin ligase, EEL-1 (Page et al., 2007). Downregulation of katanin, at an early embryogenesis stage of meiosis to mitosis transitions, is a necessity and this crucial step is majorly controlled by CUL-3 (Pintard et al., 2003). In an experimental model of the zebrafish embryo, Lnx-2a, an E3 ubiquitin ligase, balances the differentiation of exocrine cells in the early pancreatic bud, which could be achieved by fine tuning the Notch signaling via destabilization of Numb (Won et al., 2015).

**FIGURE 3:**
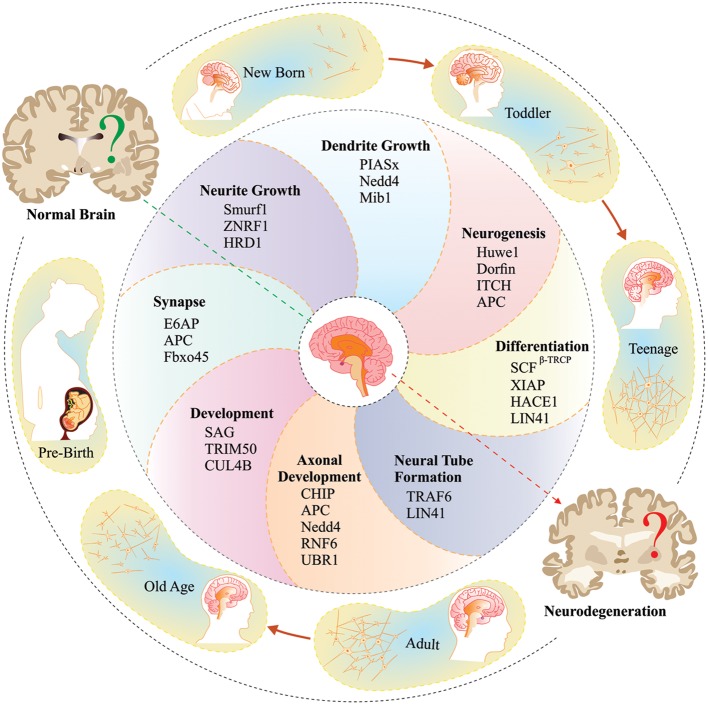
**Proposed diagrammatic representations comprehend the neurobiological roles of few E3 ubiquitin ligases associated with normal brain development.** The complex molecular mechanisms governed by these E3 ubiquitin ligases throughout the entire life span starting from learning skills (neurodevelopment) to kills (neurodegeneration) are still unknown.

Mammalian E3 ubiquitin ligases are also very crucial for the execution of various developmental processes, with striking control over neurogenesis (Stegmuller and Bonni, 2010). Hematopoietic stem cells highly express DDB1, a component of CUL4-DDB1 E3 ubiquitin ligase complex, which monitors stem cell divisions by ubiquitylation and proteasomal degradation of p53 (Gao et al., 2015). An early development transcription factor SOX-9, is ubiquitinated and degraded by Fbxw7 E3 ubiquitin ligase (Hong et al., 2016). ASB4 and ASB2/Cullin5/Rbx complexes degrade filamin B proteins, and play important roles in myogenic differentiation (Bello et al., 2009; Townley-Tilson et al., 2014); whereas, S-phase kinase-associated protein1-cullin1-F-box protein (SCF^Skp2^) complex plays important roles in differentiation of the neuronal cells (Boix-Perales et al., 2007). The functions of E3 ubiquitin ligases are required from the initiation of neuronal differentiation and up to the maturation of neurons and formation of synapses (Yi and Ehlers, 2007; Ding and Shen, 2008). Various mutations in E3 ubiquitin ligase genes and UPS dysfunction may lead to different kinds of abnormalities and disorders related to the neuronal development and functioning (Tai and Schuman, 2008; Kawabe and Brose, 2011). In **Figure 3**, we have schematically represented several reported E3 ubiquitin ligases that significantly contribute in regulation of various processes and pathways of neuronal development.

Huwe1, an upstream regulator of N-Myc-DLL3-Notch signaling pathway restricts proliferation and initiates differentiation of neuronal stem cells (Zhao et al., 2009). Dorfin also plays an important role in neurogenesis; as reported in a recent study, Dorfin^-/-^ mice show an overall decrease in the ubiquitylation level of various proteins (Park et al., 2015). Another crucial neurodevelopmental E3 ubiquitin ligase, involved in regulation of Notch signaling, is Mib, which ubiquitinate Delta followed by endocytosis (Itoh et al., 2003). XIAP plays regulatory roles in differentiation and neurogenesis, by controlling the formation of axons and dendrites; which is mediated by activation of MEK pathway (Fado et al., 2013). Other E3 ubiquitin ligases, like HACE1, and β-TRCP (SCF^β-TRCP^), also have roles in controlling neuronal differentiation, via interaction and degradation of different client proteins (Westbrook et al., 2008; Hollstein et al., 2015). Lin41-mediated terminal differentiation sets the timing of neural tube closure, and mutation of this gene results in embryonic lethality (Maller Schulman et al., 2008). Similarly, deficiency of TRAF6 causes abnormal neural tube closure and may result in exencephaly (Lomaga et al., 2000).

Hrd1, an ER stress E3 ubiquitin ligase, facilitates induced neuronal differentiation and reduced neurite outgrowths (Kawada et al., 2014); whereas, Smurf1 promotes neurite outgrowth by ubiquitylation and proteasomal degradation of RhoA (Bryan et al., 2005). ZNRF1 E3 ubiquitin ligase mediates similar positive effects on neuritogenesis by interacting with tubulin (Yoshida et al., 2009). Mib E3 ubiquitin ligase also has significant control over neuritogenesis (Choe et al., 2007), as well as dendritic morphogenesis (Smrt et al., 2010). A study on granule neurons of cerebral cortex identified a critical role of PIASx, a SUMO E3 ubiquitin ligase, in postsynaptic dendritic differentiation (Shalizi et al., 2007). Ubiquitylation-mediated degradation of Rap2A and PTEN by NEDD4-1 are crucial events in dendritic development (Kawabe et al., 2010) and axonal branching, respectively (Drinjakovic et al., 2010). RNF6 and CHIP are also involved in axonal developmental processes by affecting the degradation of their development-associated substrates, viz., LIMK1 and katenin-p60, respectively (Tursun et al., 2005; Yang et al., 2013). Further, Ubr1-mediated N-end rule protein degradation of some unknown neurite outgrowth inhibitory protein also has critical involvement in neurite outgrowth and axonal regeneration processes (Kavakebi et al., 2005). Neuronal pruning is another crucial step of neurodevelopment, which is influenced by another E3 ubiquitin ligase, i.e., cullin1 based SCF complex (Wong et al., 2013).

Cell cycle regulatory E3 ubiquitin ligase, APC/C, has also been characterized for its determining roles in neuronal morphogenesis and connectivity (Yang et al., 2010). A complex of Cdh1-APC has shown control over axonal growth and patterning (Konishi et al., 2004), while Cdc20-APC E3 ubiquitin ligase has shown regulatory effects over dendritic morphogenesis and presynaptic axonal differentiation, helping synapse formation and development of neuronal circuits (Kim et al., 2009; Yang et al., 2009). Fbxo45, an F-box containing protein, instead of interacting with SCF, forms an E3 ubiquitin ligase complex with PAM, and this complex is important for neuronal migration and synapse formation at neuromuscular junctions (Saiga et al., 2009). SAG, a RING domain protein of SCF complex targets NF1 and modulates Ras-MAPK signaling pathway to affect neuronal development (Tan et al., 2011b).

There are several E3 ubiquitin ligases, whose loss-of-function has prominent roles in one or more developmental abnormalities or disorders. CUL4B mutation may result in X-linked mental retardation (Zou et al., 2007); whereas TRIM family of E3 ubiquitin ligases have been implicated in several developmental disorders, e.g., TRIM50 in Williams–Beuren syndrome (Micale et al., 2008), Midline 1 (MID1) in Opitz syndrome (Quaderi et al., 1997), and TRIM37 in Mulibrey Nanism (Kallijarvi et al., 2002). As discussed above, most of the substrates of E3 ubiquitin ligases are regulatory proteins involved in different signaling pathways, controlling cell cycle progression, cellular metabolism, apoptosis, etc., and hence may impact development and growth of organism (Bielskiene et al., 2015). Thus, disturbances caused in the ubiquitylation mechanism due to impairment in E3 ubiquitin ligases functionalities may lead to different kinds of abnormal conditions, developmental disorders, and embryonic lethality (Huang and Dixit, 2016). Therefore, identification and characterization of methodologies that may have the ability to modulate or stabilize the activities of E3 ubiquitin ligases may prove to be a beneficial strategy in controlling the progression of various disorders (Goldenberg et al., 2010).

The overall development of the brain is affected by multiple factors, including above described environmental factors and genes participating in neurodevelopmental processes; and various disease conditions could originate either due to their dysregulation or non-functional QC system of the cells (van Loo and Martens, 2007; Bale et al., 2010). Once neurodevelopmental period reaches its completion, several internal and external factors start presenting threats and challenges before neurons (Cannon and Greenamyre, 2011; Chin-Chan et al., 2015). A progressive decline in function of cellular defense mechanisms may drive organisms toward aging and result in late-age neurodegeneration (Pettegrew et al., 2000; Crawley, 2006). Age-related neurodegeneration is directly or indirectly associated with various diseases like Alzheimer’s, Parkinson’s, Huntington’s, ALS, and many more (Hung et al., 2010; Niccoli and Partridge, 2012). Roles of E3 ubiquitin ligases and the cellular PQC system have largely been explored and their deficiency has been attributed to several prominent diseases, associated with protein misfolding (Ardley and Robinson, 2004; Chhangani et al., 2012). Interestingly, several reports have shown that a well-regulated proteolysis, mediated by E3 ubiquitin ligases, is an important measure to ensure an error-proof development of organisms, which also holds true for plants (Vierstra, 1996; Sullivan et al., 2003).

There are number of E3 ubiquitin ligases, which have been identified in plants, affecting major developmental pathways, including perception of hormones and their respective signaling (Schwechheimer and Schwager, 2004; Stone and Callis, 2007). Abscisic acid signaling is an important regulator of plant growth and development at various stages, including seed dormancy and germination, embryonic development and reproduction, and vegetative growth, etc. (Wasilewska et al., 2008). Several E3 ubiquitin ligases have been identified for their roles in regulation and maintenance of abscisic acid biosynthesis and signaling pathways (Liu and Stone, 2011). CHIP (Luo et al., 2006), Keep on Going (KEG) (Stone et al., 2006), ABI3-interacting protein 2 (AIP2) (Zhang et al., 2005), and senescence-associated E3 ubiquitin ligase 1 (SAUL1) (Raab et al., 2009) are major regulators of ubiquitination and proteasomal degradation mediated control of the plant development. Auxins are the hormones that are implicated in plants root development. Transport inhibitor response 1 (TIR1), XB3 ortholog 2 in *Arabidopsis thaliana* (XBAT32), and seven-in-absentia of *A. thaliana* 5 (SINAT5) E3 ubiquitin ligases actively regulate auxin signaling and formation of lateral roots (Xie et al., 2002; Nodzon et al., 2004; Tan et al., 2007). Several other signaling pathways and mechanisms have also been explored in detail, where E3 ubiquitin ligases are extensively involved in development of plants (Moon et al., 2004; Stone and Callis, 2007; Chen and Hellmann, 2013).

It is now well-known that the environmental toxicants affect both, the processes of neurodevelopment, as well as neurodegeneration; and thus may increase the risk of brain-related disorders multiple folds (Gressens et al., 2001; Landrigan et al., 2005; Aschner and Costa, 2015). So, it is worthy to ask questions of how we can protect our cellular systems from toxicities and damages produced by these external factors. Considering the enormous potential of E3 ubiquitin ligases in the modulation of functions of intracellular proteins and regulation of cellular processes, it is obvious to question if we can modulate these mechanisms and pathways exogenously. To exploit therapeutic potential of E3 ubiquitin ligases, several attempts have been made to devise therapeutic strategies against various diseases.

## E3 Ubiquitin Ligases: Possible Emerging Molecular Functions, Targets, and Therapeutic Applications

Past few years of research have documented several efforts toward the development of therapeutic strategies by exploiting druggability of these E3 ubiquitin ligases, by using many natural and synthetic small molecules, having modulating effects over these ligases. As shown in **Table 1**, we have summarized various available reports so far. Previous studies on natural molecules, like curcumin or diferuloylmethane, extracted from the rhizomes of herb *Curcuma longa* induces apoptosis in cancerous cells by inhibiting the Cdc27/APC3 component of APC, which is important for its ubiquitylation function (Lee and Langhans, 2012). Trehalose, a disaccharide has been reported to increase levels of CHIP and further induce autophagy in CHIP mutation related ataxia in fibroblast cells (Casarejos et al., 2014). A similar effect on the level of CHIP and autophagy induction has recently been reported for lanosterol, an intermediate compound of cholesterol biosynthesis (Upadhyay et al., 2017). MLN4924, an inhibitor of E3 ubiquitin ligase activity of SAG-SCF, has shown a promising effect in sensitizing leukemia cells toward the effects of retinoic acid (Tan et al., 2011a). It has also been shown that blocking the enzymatic activity of Nedd4, thereby modulating the budding of viruses, filoviruses, arenaviruses. and rhabdoviruses, may help in the development of a novel class of antiviral therapy (Han et al., 2014). A proteasome inhibitor, MG132 has also been shown to increase the level of Ubr1, and this may act as a useful therapeutic strategy in pathologies caused due to reduced amount of Ubr1, like pancreatic dysfunctions and mental abnormalities, characteristics of Johanson-Blizzard syndrome (Zenker et al., 2005). Similar effects of increasing E3 ubiquitin ligase activity have also been demonstrated for ZNRF1, in response to treatment of 6-hydroxydopamine (Wakatsuki et al., 2015).

**Table 1:** Summarizes E3 ubiquitin ligases involved in process of neurodevelopment and promising molecules that have been shown to modulate their activity and retain possible therapeutic potential.

Modulators/Stress inducing agents	E3 ubiquitin ligases	Molecular mechanism	Reference
Curcumin	APC	Inhibits by binding to APC leading to cell cycle arrest and apoptosis.	Lee and Langhans, 2012
Trehalose	CHIP	Increases expression level of CHIP.	Casarejos et al., 2014
Macrocyclic *N*-methyl-peptides	E6AP	Inhibits the E6AP catalyzed polyubiquitination of peroxiredoxin 1 and p53.	Yamagishi et al., 2011
Ubiquitin variant	HACE1	Reduces activity of HACE1 by inhibiting ubiquitin transfer to its substrate Rac1.	Zhang et al., 2016
Thapsigargin/Tunicamycin	HRD1	Increases expression level of HRD1 at mRNA and protein levels by induction of endoplasmic reticulum stress.	Kaneko et al., 2007
Doxycycline	Huwe1	Decreases expression of Huwe1 which in turn stabilizes MYC-associated protein MIZ1 causing inhibition of MYC function.	Peter et al., 2014
Compounds 4 and 5	Nedd4	Inhibits Nedd4-PPxY interaction thereby blocking egress of RNA viruses.	Han et al., 2014
PF-03084014	Numb	Reverses docetaxel mediated decreased expression of Numb, an endogenous notch inhibitor producing an increased cytotoxic effect of docetaxel.	Zhang et al., 2013
Cisplatin	RNF6	Inhibits RNF6 expression level in A549 cell line as compared to A549 cisplatin resistant cells (A549/CDDP cells).	Qin et al., 2012
MLN4924	SAG	Impede SAG activity by blocking its cullin neddylation which can sensitize leukemia cell lines HL-60 and KG-1 to retinoic acid.	Tan et al., 2011a
CpdA	SCF	Interferes with SCF (Skp2) ligase function causing p27 mediated cell cycle arrest and activation of autophagy.	Chen et al., 2008
A01 and A17	Smurf1	Inhibits Smurf1 mediated Smad1/5 proteasomal degradation by reducing its ubiquitination and increases bone morphogenetic protein (BMP-2) signal responsiveness.	Cao et al., 2014
Bortezomib	TRAF6	Decreases expression of TRAF6 at both protein and mRNA level leading to inhibition of osteoclasts maturation and function in peripheral blood mononuclear cells (PBMCs) of multiple myeloma patients.	Hongming and Jian, 2009
MG132	UBR1	Induces upregulation of ubr1 through ubiquitin-proteasome pathway.	Yu et al., 2010
XIAP antagonist	XIAP	Inhibits by binding to the baculovirus IAP repeat 3 (BIR3) domain of XIAP.	Seigal et al., 2015
6OHDA	ZNRF1	Induces ZNFR1 E3 ligase activity by EGFR-mediated phosphorylation.	Wakatsuki et al., 2015

To devise the molecular therapeutics that can be used as potential strategies for modulating the functions and activities of various E3 ubiquitin ligases in treating multiple disorders, extensive studies are required, which can characterize these lead molecules for their efficacy, side effects, safety and pharmacological profiles. Although few drugs, such as Nutlin-3, a Mdm2-p53 interaction inhibitor, is used for treating pathology of cancer(Vassilev et al., 2004), but it is still in clinical trials and has also shown few side effects in the treatment of retinoblastoma (Secchiero et al., 2011). Development of ubiquitin variant probes having the abilities to target the ubiquitylation functions of E3 ubiquitin ligase can also prove to be an effective strategy in disease treatment (Zhang et al., 2016). Finding out solutions to these problems will help significantly in bringing these drugs or small molecules from basic research to the level of therapeutic applications.

The need of cost-effective therapies that have minimal side effects and which can reach up to every individual is not yet successfully fulfilled, and thus extensive exploration of natural and synthetic compounds will enable us to devise useful, and effective strategy to prevent and cure such diseases (Tsukamoto, 2016; Collins et al., 2017). The above mentioned small molecules are merely a fraction of research, which could be considered for upcoming strategies to treat fatal neurodegenerative disorders, cancer or other complex diseases, but there still remains a need for more research to be done (Eldridge and O’Brien, 2010; Gurevich and Gurevich, 2014; Skaar et al., 2014). Targeting specific E3 ubiquitin ligase for the treatment of particular disease is not an easy way to exercise in therapeutic applications, as these E3 ubiquitin ligases get involved in multiple pathways simultaneously. Therefore, there remains an immense need of understanding complete functionality and specificity, which these E3 ubiquitin ligases could provide in order to get the maximum beneficial outcome for future drug development strategies.

## Key Questions and Future Prospective

All proteins originate through a common mechanism of synthesis, but they largely differ in their degradatory pathways (Ciechanover, 2005; Tai and Schuman, 2008). Neuronal cells, which have unique characteristics with a distinct morphology, critically require a well-regulated system of synthesis and degradation of proteins at the time of development, as well as in later life stages (Yi and Ehlers, 2007; Takano et al., 2015). They maintain a polar shape, in order to develop a functional neuronal circuitry (Tahirovic and Bradke, 2009). It is now evident from multiple studies that cellular proteolytic machinery is exquisitely implicated in the formation of synapses and neuronal circuitry, by maintaining the polarity of neurons (Murphey and Godenschwege, 2002; Bingol and Schuman, 2005). Several crucial E3 ubiquitin ligases, viz., TRIM2, TRIM46, and FBXO31-SCF, have been found to have a regulatory control over migration, polarization and synapse formation (Khazaei et al., 2011; Vadhvani et al., 2013; van Beuningen et al., 2015). A number of reports postulate the decisive involvement of PQC system in the maintenance of cellular proteostasis, and thus in ameliorating the toxicities generated by the accumulation of misfolded proteins in late-age. But, in past many years, indispensable roles of E3 ubiquitin ligases in proteasome-mediated proteolysis of crucial substrate proteins, which play important roles in several pathways of neuronal development, have been investigated in detail (Segref and Hoppe, 2008; Tai and Schuman, 2008; Yamada et al., 2013).

Major neurodevelopmental abnormalities are genetically complex disorders, and are caused by multifactorial aberrations in the developing brain. A very genuine challenge is to understand the precision of molecular pathomechanisms involved in NDDs. The future research on brain disorders must address the importance of QC E3 ubiquitin ligases in regulation of major signaling pathways that could link critical substrates involved in NDDs and neurodegenerative diseases. Dysregulation of protein synthesis, comprising proper folding of proteins, has found to be involved in induction of several stress-response pathways. Accumulation of abnormal proteins may affect the synaptic functions, neuronal connectivity and can also alter normal brain functions. Improper protein translation with a lack of cellular protein QC mechanism is the most prominent factor, which disturbs normal cellular proteostasis and can cause genetic diseases, neurodegeneration, and aging.

So far, we have not understood completely how cellular protein QC machinery contributes to the regulation of those genes, which are important for brain development and normal functions of brain throughout lifespan. Despite many findings in basic human molecular genetics reveal the underlying causes and thus enable us to challenge the occurrence of NDDs and neurodegeneration; still a conclusive solution to modulate the large number of genes and their end product proteins in a favorable manner at early and old age, is a real challenge in development of successful therapeutic strategies. It will be interesting in upcoming research to find out how QC E3 ubiquitin ligases control synapse formation and axon branching during developmental period. How these QC E3 ubiquitin ligases regulate protein synthesis, and prevent accumulation of toxic and non-functional proteins in neurons at later stages of life? Hence, in future, it is important to understand how we can tightly regulate cellular PQC mechanisms during early and later phases of life with the help of known cellular quality control markers such as QC E3 ubiquitin ligases.

## Author Contributions

VJ, AU, AA, and RM, executed complete drawing of figures, NA and AP provided critical inputs. AM formulated the entire concept of manuscript and designed the initial draft of figures. All authors reviewed the manuscript.

## Conflict of Interest Statement

The authors declare that the research was conducted in the absence of any commercial or financial relationships that could be construed as a potential conflict of interest.

## Abbreviations

ADHD: attention-deficit hyperactivity disorder
AIP4: atrophin-1-interacting protein 4
ALS: amyotrophic lateral sclerosis
APC/C: anaphase-promoting complex/cyclosome
APP: amyloid precursor protein
ASB4: ankyrin repeat and SOCS box containing protein 4
ASD: autism spectrum disorder
β-TRCP: β-transducin repeat-containing protein
CHIP: Carboxy-terminus of Hsc70-interacting protein
CNTNAP2: contactin-associated protein-like 2
CRL: cullin-RING like ubiquitin ligases
DDB1: DNA damage-binding protein 1
DISC1: disrupted-in-schizophrenia 1
E6-AP: E6-associated protein
EEL-1: enhancer of EfL-1 mutant phenotype
Fbxw: F-box and WD40 domain
FMR1: fragile X mental retardation
HACE1: HECT domain and ankyrin repeat containing E3 ubiquitin ligase 1
HECT: homologous to the E6-AP carboxyl terminus
ID: Intellectual disabilities
LIMK-1: LIM domain kinase 1
LIN41: lineage variant 41
Lnx: ligand of Numb protein-X
LTP: long-term potentiation
MAPK: Mitogen-activated protein kinase
MECP2: methyl-CpG-binding protein 2
MEK: MAPK ERK kinase
MGRN1: mahogunin RING finger protein 1
Mib1: mind bomb 1
MID1: midline-1
NDD: neurodevelopmental disorders
NDE1: nuclear distribution E homolog 1
NEDD4: neural precursor cell expressed developmentally down-regulated protein 4
NF-1: Neurofibromatosis type 1
NLGN: neuroligin
NRF2: nuclear factor erythroid 2-related factor 2
PAM: protein associated with Myc
PIAS: protein inhibitor of activated STAT
PINK1: PTEN-induced putative kinase 1
PMS: Phelan-McDermid syndrome
PQC: protein quality control
PTEN: phosphatase and tensin homolog
Rap2A: Ras-related protein 2A
RING: really interesting new gene
RNF: RING finger protein
SAG: sensitive to apoptosis gene
SCF: S-phase kinase-associated protein1 (SKP1)-cullin1 (CUL1)-F-box protein
SCZ: schizophrenia
SHANK3: SH3 and ankyrin domain-containing protein 3
SK2: small-conductance calcium-activated potassium channel 2
SKN-1: skinhead-1
SOD1: superoxide dismutase 1
SOX9: SRY homeobox 9
SUMO: small ubiquitin-like modifier
TDP43: transactive response DNA-binding protein-43
TRAF: TNF receptor associated factor
TRIM: tripartite motif
UBE3A: ubiquitin protein ligase E3A
UPS: ubiquitin-proteasome system
ZNRF1: zinc and ring finger 1.
